# Clinical standards for drug-susceptible TB in children and adolescents

**DOI:** 10.5588/ijtld.23.0085

**Published:** 2023-08-01

**Authors:** S. S. Chiang, S. M. Graham, H. S. Schaaf, B. J. Marais, C. C. Sant’Anna, S. Sharma, J. R. Starke, R. Triasih, J. Achar, F. Amanullah, L. Y. Armitage, R. B. Aurilio, W. C. Buck, R. Centis, C. Chabala, A. T. Cruz, A.-M. Demers, K. du Preez, A. Enimil, J. Furin, A. J. Garcia-Prats, N. E. Gonzalez, G. Hoddinott, P. Isaakidis, D. Jaganath, S. K. Kabra, B. Kampmann, A. Kay, I. Kitai, E. Lopez-Varela, E. Maleche-Obimbo, F. Mestanza Malaspina, J. Niederbacher Velásquez, J. J. C. Nuttall, J. N. Oliwa, I. Orozco Andrade, C. M. Perez-Velez, H. Rabie, J. A. Seddon, M. P. Sekadde, A. Shen, A. Skrahina, A. Soriano-Arandes, A. P. Steenhoff, M. Tebruegge, M. A. Tovar, B. Tsogt, M. M. van der Zalm, H. Welch, G. B. Migliori

**Affiliations:** 1 Division of Pediatric Infectious Diseases, Department of Pediatrics, Alpert Medical School of Brown University, Providence, RI; 2 Center for International Health Research, Rhode Island Hospital, Providence, RI, USA; 3 Department of Paediatrics, University of Melbourne, Melbourne, VIC; 4 Burnet Institute, Melbourne, VIC, Australia; 5 Desmond Tutu TB Centre, Department of Paediatrics and Child Health, Faculty of Medicine and Health Sciences, Stellenbosch University, Cape Town, South Africa; 6 Department of Paediatrics and Child Health and the Sydney Infectious Diseases Institute (Sydney ID), Sydney, NSW; 7 Department of Infectious Diseases, The Children’s Hospital at Westmead, Sydney, NSW, Australia; 8 Department of Paediatrics, Universidade Federal do Rio de Janeiro, Rio de Janeiro, RJ, Brazil; 9 Department of Paediatrics, National Institute of Tuberculosis and Respiratory Diseases, New Delhi, India; 10 Department of Pediatrics, Baylor College of Medicine, Houston, TX; 11 Section of Infectious Diseases, Texas Children’s Hospital, Houston, TX, USA; 12 Department of Paediatrics, Faculty of Medicine, Public Health and Nursing, Universitas Gadjah Mada/Dr Sardjito Hospital, Yogyakarta, Indonesia; 13 Department of Global Public Health, Karolinska Institutet, Stockholm, Sweden; 14 Department of Paediatrics, The Indus Hospital and Health Network, Karachi; 15 Department of Paediatrics, The Aga Khan University Hospital, Karachi, Pakistan; 16 Heartland National TB Center, University of Texas Health Science Center at Tyler, San Antonio, TX, USA; 17 Department of Paediatrics, Instituto de Puericultura e Pediatria Martagão Gesteira, Universidade Federal do Rio de Janeiro, Rio de Janeiro, RJ; 18 Department of Paediatrics, Universidade do Estado do Rio de Janeiro, Rio de Janeiro, RJ, Brazil; 19 Department of Pediatrics, University of California Los Angeles David Geffen School of Medicine, Los Angeles, CA, USA; 20 Respiratory Diseases Clinical Epidemiology Unit, Istituti Clinici Scientifici Maugeri, Istituto di Ricovero e Cura a Carattere Scientifico, Tradate, Italy; 21 School of Medicine, Department of Paediatrics and Child Health, University of Zambia, Lusaka; 22 Children’s Hospital, University Teaching Hospitals, Lusaka, Zambia; 23 Division of Microbiology, Department of Laboratory Medicine, CHU Sainte-Justine, Montreal, Canada; 24 Department of Child Health, Kwame Nkrumah University of Science and Technology, Kumasi; 25 Department of Child Health, Komfo Anokye Teaching Hospital, Kumasi, Ghana; 26 Department of Global Health and Social Medicine, Harvard Medical School, Boston, MA; 27 Division of Infectious Diseases, Department of Medicine, Case Western Reserve University, Cleveland, OH; 28 Department of Pediatrics, University of Wisconsin-Madison, Madison, WI, USA; 29 División Neumotisiología, Hospital de Niños Pedro de Elizalde, Buenos Aires; 30 Dirección General de Posgrado, Facultad de Medicina, Universidad de Buenos Aires, Buenos Aires, Argentina; 31 Southern Africa Medical Unit (SAMU), Médecins Sans Frontières, Cape Town, South Africa; 32 Clinical and Molecular Epidemiology Unit, Department of Hygiene and Epidemiology, University of Ioannina School of Medicine, Ioannina, Greece; 33 Division of Pediatric Infectious Diseases, Department of Pediatrics, University of California San Francisco, San Francisco, CA, USA; 34 Department of Paediatrics, All India Institute of Medical Sciences, New Delhi, India; 35 Charite Centre for Global Health, Charite Universitatsmedizin Berlin, Berlin, Germany; 36 Clinical Research Department, London School of Hygiene & Tropical Medicine, London, UK; 37 Department of Paediatrics, Hospital for Sick Children, Toronto, ON; 38 Department of Pediatrics, University of Toronto, Toronto, ON, Canada; 39 Hospital Clínic and ISGlobal, Universitat de Barcelona, Barcelona, Spain; 40 Centro de Investigação em Saúde de Manhiça (CISM), Manhiça, Mozambique; 41 Department of Paediatrics & Child Health, University of Nairobi, Nairobi, Kenya; 42 Department of Paediatrics, Hospital San Bartólome, Lima; 43 Red Peruana de Tuberculosis Pediátrica, Dirección de Prevención y Control de Tuberculosis, Ministerio de Salud, Lima, Perú; 44 Department of Paediatrics, Universidad Industrial de Santander, Bucaramanga; 45 Board of Directors, Asociación Colombiana de Neumología Pediátrica, Bogotá, Colombia; 46 Paediatric Infectious Diseases Unit, Department of Paediatrics and Child Health, University of Cape Town, Cape Town, South Africa; 47 Faculty of Health Sciences, Department of Paediatrics and Child Health, The University of Nairobi, Nairobi; 48 Health Services Unit, Kenya Medical Research Institute-Wellcome Trust Research Programme, Nairobi, Kenya; 49 Center of Diagnosis and Integral Treatment for Tuberculosis, Servicios Médicos de la Frontera, Juárez; 50 Medical Coordination, Juntos Binational Tuberculosis Project, Juárez, México; 51 Division of Infectious Diseases, Department of Internal Medicine, University of New Mexico, Albuquerque, NM, USA; 52 Department of Paediatrics and Child Health, Faculty of Medicine and Health Sciences, Stellenbosch University, Cape Town, South Africa; 53 Department of Infectious Disease, Imperial College London, London, UK; 54 National TB and Leprosy Program, Ministry of Health, Kampala, Uganda; 55 Beijing Paediatric Research Institute, National Centre for Children’s Health, Beijing Children’s Hospital, Capital Medical University, Beijing; 56 Pediatric Research Institute, Henan Children’s Hospital, Zhengzhou Children’s Hospital, Zhengzhou, China; 57 Clinical Department, The Republican Scientific and Practical Centre for Pulmonology and TB, Minsk, Belarus; 58 Paediatric Infectious Diseases and Immunodeficiencies Unit, Hospital Universitari Vall d’Hebron, Barcelona; 59 Infection and Immunity in Children, Vall d’Hebron Research Institute, Barcelona, Spain; 60 Global Health Center and Department of Pediatrics, Children’s Hospital of Philadelphia, Philadelphia, PA, USA; 61 Department of Paediatric & Adolescent Health, Faculty of Medicine, University of Botswana, Gaborone, Botswana; 62 Department of Infection, Immunity & Inflammation, University College London, Great Ormond Street Institute of Child Health, London, UK; 63 Department of Paediatrics, Klinik Ottakring, Wiener Gesundheitsverbund, Vienna, Austria; 64 Socios En Salud Sucursal Perú; 65 Escuela de Medicina, Facultad de Ciencias de la Salud, Universidad Peruana de Ciencias Aplicadas, Lima, Perú; 66 Research and Innovation, Mongolian Anti-TB Coalition, Ulaanbaatar, Mongolia; 67 Department of Paediatrics, The University of Papua New Guinea School of Medicine and Health Sciences, Port Moresby, Papua New Guinea

**Keywords:** diagnosis, treatment, meningeal tuberculosis, miliary tuberculosis, tuberculosis infection, HIV

## Abstract

**BACKGROUND:**

These clinical standards aim to provide guidance for diagnosis, treatment, and management of drug-susceptible TB in children and adolescents.

**METHODS:**

Fifty-two global experts in paediatric TB participated in a Delphi consensus process. After eight rounds of revisions, 51/52 (98%) participants endorsed the final document.

**RESULTS:**

Eight standards were identified: Standard 1, Age and developmental stage are critical considerations in the assessment and management of TB; Standard 2, Children and adolescents with symptoms and signs of TB disease should undergo prompt evaluation, and diagnosis and treatment initiation should not depend on microbiological confirmation; Standard 3, Treatment initiation is particularly urgent in children and adolescents with presumptive TB meningitis and disseminated (miliary) TB; Standard 4, Children and adolescents should be treated with an appropriate weight-based regimen; Standard 5, Treating TB infection (TBI) is important to prevent disease; Standard 6, Children and adolescents should receive home-based/community-based treatment support whenever possible; Standard 7, Children, adolescents, and their families should be provided age-appropriate support to optimise engagement in care and clinical outcomes; and Standard 8, Case reporting and contact tracing should be conducted for each child and adolescent.

**CONCLUSION:**

These consensus-based clinical standards, which should be adapted to local contexts, will improve the care of children and adolescents affected by TB.

## Abstract (fr)

**CONTEXTE:**

Les normes cliniques décrites ci-dessus visent à fournir des orientations pour le diagnostic, le traitement et la prise en charge de la TB pharmacosensible chez les enfants et les adolescents.

**MÉTHODES:**

Cinquante-deux experts mondiaux de la TB pédiatrique ont participé à un processus de consensus Delphi. Après huit séries de révisions, 51/52 (98%) des participants ont approuvé le document final.

**RÉSULTATS:**

Huit normes ont été identifiées: Norme 1, L’âge et le stade de développement sont des considérations essentielles dans l’évaluation et la prise en charge de la TB; Norme 2, Les enfants et les adolescents présentant des symptômes et des signes de TB doivent faire l’objet d’une évaluation rapide, et le diagnostic et l’instauration du traitement ne doivent pas dépendre de la confirmation microbiologique; Norme 3, L’instauration du traitement est particulièrement urgente chez les enfants et les adolescents atteints de méningite tuberculeuse présumée et de TB disséminée (miliaire); Standard 4, Les enfants et les adolescents doivent être traités avec un régime approprié basé sur le poids; Standard 5, Le traitement de l’infection tuberculeuse (TBI) est important pour prévenir la maladie; Standard 6, Les enfants et les adolescents doivent recevoir un soutien thérapeutique à domicile ou dans la communauté chaque fois que possible; Standard 7, Les enfants, les adolescents et leurs familles doivent recevoir un soutien adapté à leur âge afin d’optimiser l’engagement dans les soins et les résultats cliniques; et Standard 8, La déclaration des cas et la recherche des contacts doivent être effectuées pour chaque enfant et chaque adolescent.

**CONCLUSION:**

Ces normes cliniques consensuelles, qui devraient être adaptées aux contextes locaux, amélioreront la prise en charge des enfants et des adolescents atteints de TB.

Globally, an estimated 44.7 million children (0–9 years old) and 125.3 million adolescents (10–19 years old) are infected with *Mycobacterium tuberculosis*.^1^ Of these, approximately 1.8 million develop TB disease each year. The WHO estimates that deaths due to TB numbered 216,570 in children and young adolescents aged <15 years in 2021 and 60,000 in adolescents aged 15–19 years in 2019.^2^,^3^ An estimated 96% of all TB deaths in children occur in those who never received treatment.^4^ In 2018, the United Nations High-Level Meeting (UNHLM) on TB set a TB disease treatment target of 3.5 million children between 2018 and 2022; however, only 1.9 million (54%) had been treated by the end of 2021. The UNHLM also set a target to treat TB infection (TBI) in 4.0 million household contacts under 5 years of age with TB preventive therapy (TPT); only 1.6 million (40%) had been treated by the end of 2021. Treatment of TBI in household contacts aged ≥5 years was achieved in only 3% of the set target.^2^ These figures highlight that a greater global effort is needed to identify children and adolescents with TBI and TB disease and to ensure treatment access and completion.

TB is both preventable and curable. Strategies to prevent TB disease include vaccination of infants with bacille Calmette Guérin (BCG) in high-incidence settings, timely diagnosis and treatment of infectious (usually older adolescent and adult) TB patients, and providing TPT to children and adolescents in close contact with infectious TB patients after excluding TB disease. To reduce morbidity and mortality, early diagnosis and prompt treatment initiation are needed and can be achieved through an increased awareness of TB in children and adolescents among healthcare workers, caregivers and other members of the community; a high index of suspicion for TB; knowledge on how to diagnose TB in both passive (presenting with symptoms) and active (contact tracing) case-finding; and knowledge on how to effectively treat children and adolescents with non-severe and severe TB.

The IJTLD Clinical Standards for Lung Health complement existing WHO and other guidelines and integrate their recommendations to provide a specific clinical focus.^5^–^8^ The standards require context-specific implementation in various settings and are based on the best available data, although evidence in some areas remains limited. Differences in capacity and access to technology mean that some settings or services may not be able to meet all standards that define the aspirational goal and optimal standard of care. This consensus document describes clinical standards for the care of drug-susceptible TB in children and adolescents, with subdivision into more granular age groups that reflect relevant biological and psychosocial differences.^9^,^10^ The group definitions are:

Children: 0 to <10 years of age

- Infants: <1 year of age
- Young children: <5 years of age (including infants)
- Older children: 5 to <10 years of age

Adolescents: 10 to <20 years of age

- Young adolescents: 10 to <15 years of age
- Older adolescents: 15 to <20 years of age

## AIM OF THE CLINICAL STANDARDS

The following standards were identified:

1. Age and developmental stage are critical considerations in the assessment and management of TB (Standard 1);
2. Children and adolescents with symptoms and signs compatible with TB disease should undergo prompt evaluation using available diagnostic tools, including chest X-ray (CXR) and microbiological assays. The diagnosis of TB disease and treatment initiation should not be conditional on microbiological confirmation (Standard 2);
3. TB treatment initiation is particularly urgent in children and adolescents with presumptive TB meningitis (TBM) and disseminated (miliary) TB (Standard 3);
4. Children and adolescents with TB disease should be treated with an appropriate weight-based regimen as recommended by the WHO and/or national guidelines (Standard 4);
5. Evaluation and treatment of TBI is important to prevent disease, especially for those at high risk of disease progression following infection (Standard 5);
6. Children and adolescents treated for *M. tuberculosis* infection or TB disease should receive home-based/community-based treatment support whenever possible (Standard 6);
7. Children, adolescents and their families should be provided adequate, age-appropriate support to optimise engagement in care, treatment adherence, clinical outcome, and the detection and management of adverse drug reactions and post-TB sequelae (Standard 7);
8. Case notification by health facilities, recording and reporting by in-country health authorities to the WHO, and contact tracing should be conducted for each child and adolescent with TB (Standard 8).

We also developed a series of suggestions for future research to fill gaps in our current understanding.

## METHODS

Sixty experts in child and adolescent TB were invited to contribute. The 56 who agreed to participate were asked to evaluate an initial draft of eight standards developed by the coordination team (SSC, SMG, HSS, BJM, CSA, SS, JRS, RT) using a Delphi process. The 52 experts who participated in the consensus process represented all six WHO regions and 31 countries, 17 of which rank among the WHO-designated high-burden countries for TB, TB-HIV and/or rifampicin-resistant/multidrug-resistant TB (RR/MDR-TB).^11^ The final panel included self-identified TB clinicians (*n* = 44), public health professionals/policy makers (*n* = 10), researchers (*n* = 36), epidemiologists (*n* = 7), one microbiologist and one social scientist (respondents could identify as more than one category). A five-point Likert scale was used (5: high agreement; 1: low agreement). In the first Delphi round, the median value was 5.0 for all standards. Based on this substantial agreement, the coordination team drafted a document, which then underwent eight rounds of revision. The final version was approved by consensus (agreement: 51/52, 98%).

## STANDARD 1

**Age and developmental stage are critical considerations in the assessment and management of TB.**

The risk of progression from TBI to TB disease changes substantially through infancy, childhood and adolescence. Because of their immunological immaturity, infants and young children have the highest risk of progression to disease, including disseminated TB, of any age group.^12^ This progression can occur rapidly, sometimes within several weeks. In immunocompetent individuals, the risk reaches a nadir between ages 5–9 years and then rises again in adolescence, with a second peak in late adolescence and early adulthood.^13^,^14^ TB incidence is higher in females in mid-adolescence and then becomes substantially higher in males by early adulthood.^15^,^16^

### Clinical presentation and outcomes

In children, pulmonary TB (PTB) is often subclinical early in the course. At symptom onset, PTB may be constitutional or present as manifestations of intrathoracic lymphadenopathy and its complications. This type of TB is mainly paucibacillary, often cannot be confirmed microbiologically and is rarely transmissible. During adolescence, the clinical ‘phenotype’ of PTB transitions to adult-type disease, with more parenchymal destruction, including cavitation and risk of transmissibility.^13^ These changes in PTB ‘phenotype’ may not apply to immunocompromised adolescents; for instance, an inverse association between HIV coinfection and lung cavitation in older adolescents has been reported.^16^ Young children and immunocompromised individuals have an elevated risk of TBM and disseminated TB, which are associated with high mortality.^13^,^17^ The high risk of severe disease in young children, as well as diagnostic challenges that often lead to missed diagnosis, are why this age group accounts for an estimated 80% of paediatric TB deaths.^4^ Neonatal BCG vaccination reduces but does not eliminate this age-related risk of disseminated disease and death from TB.^18^,^19^ Other forms of extrapulmonary TB (EPTB; such as pleural or osteoarticular TB) are more common in older children and adolescents.^20^–^22^ In some settings, gender impacts diagnosis and outcome. In India, higher TB mortality among female children and adolescents has been attributed to delays in seeking medical attention for girls.^23^

### Microbiological and clinical diagnosis

Microbiological confirmation of PTB is challenging in young children because they usually cannot expectorate sputum and generally have paucibacillary disease. Even with rigorous specimen collection and laboratory methods, only 30–40% of children with PTB are culture-positive using current methods.^24^ Laboratory confirmation rates increase with age as the clinical ‘phenotype’ transitions to that of adult-type pulmonary TB. History of exposure to an infectious TB patient is valuable for clinical diagnosis, but it becomes less sensitive with increasing age as the potential range of settings for exposure and infection expand from the household to the wider community.^13^,^25^

### Person-centred care and treatment support

Healthcare services and treatment support must be tailored to developmental stage. Young children are wholly dependent on their caregivers, to whom patient education should be targeted. Older children should be engaged in discussions about their diagnosis and treatment, although the degree of involvement will depend on the child’s interest and understanding.^26^ Adolescents have unique healthcare needs as they transition from dependence to autonomy. They may want to make decisions regarding their care, yet still require support from adult caregivers as they acquire the skills and knowledge needed to navigate health services and complete treatment successfully.^27^ Barriers to adherence must be addressed for all ages, but especially for adolescents, who have higher rates of loss from TB treatment and poor adherence, particularly when they are also living with HIV.^28^ Consensus recommendations for providing person-centred care for adolescents with TB have been published.^3^

## STANDARD 2

**Children and adolescents with symptoms and signs compatible with TB disease should undergo prompt evaluation using available diagnostic tools, including chest X-ray and microbiological assays. The diagnosis of TB disease and treatment initiation should not be conditional on microbiological confirmation.**

Symptoms and signs, which depend on site of disease, are used to identify presumptive TB. Typical TB-related symptoms and signs (persistent cough, fever, fatigue/reduced playfulness and poor weight gain/weight loss) are well described. However, the presentation is not always ‘typical’, especially in young children and those immunocompromised by medical conditions or medications. Moreover, particularly in these two groups, there is substantial overlap in presentation with other diseases, including other lower respiratory tract infections (e.g., pneumonia), malnutrition or bacterial/viral meningoencephalitis. Table 1 gives the presentations of PTB and common forms of EPTB. Once a child or adolescent is presumed to have TB, the approach to diagnosis includes clinical, radiographic and microbiological assessments. The plotting of growth curves is critical for clinical evaluation and assessment of nutritional status. A positive immunological test that is evidence of TBI can be valuable, especially when there is no known or unclear history of exposure to TB. In addition to the tuberculin skin test (TST; Mantoux) and interferon-gamma release assays (IGRAs), *M. tuberculosis*-specific antigen-based skin tests (TBSTs) are now available and recommended by the WHO.^29^ These assays are more specific than traditional TSTs; however, all tests of infection have limited sensitivity in immunocompromised individuals, including those with severe TB disease or severe malnutrition, and cannot distinguish between infection and TB disease. HIV testing should be routinely offered after counselling and informed consent. CXR is an important part of the diagnostic process, especially since microbiological assays have low sensitivity for the detection of *M. tuberculosis* in children and young adolescents. A lateral view is recommended, particularly in pre-pubertal children, in whom the visualisation of hilar lymphadenopathy supports the diagnosis of PTB. Radiological changes in PTB and their diagnostic specificity vary, as detailed elsewhere,^30^ while the severity of changes is an important determining factor for duration of treatment (Table 2).

**Table 1:** Common symptoms and signs with different types of TB

Type of TB	Symptoms/signs	Comments
All types of TB	Weight loss/failure to thrive Fever Loss of appetite Reduced playfulness in young children Chronic fatigue in older children/adolescents Night sweats (drenching)	Use of growth chart important to detect weight loss/failure to thrive. Many of these symptoms appear gradually and subtly. Encourage caregivers to compare the child to other children in the home or to prior behaviour a year or so before
Pulmonary TB	Chronic non-remitting cough >14 days, although can be of acute onset in young and/or immunocompromised children Persistent wheeze or stridor Signs of lower respiratory tract infection, Haemoptysis, mainly in adolescents with cavitary pulmonary TB	Wheeze can be monophonic and unilateral and does not improve with bronchodilation. Signs of lower respiratory tract infection include dullness on percussion; bronchial breathing, crackles, unilateral decreased air entry on auscultation. Signs are often minimal or out of keeping with the clinical and radiographic findings
Pleural effusion	Chest pain, may cause acute shortness of breath if large Stony dullness, decreased air entry	Large pleural effusions occur mainly in older children and adolescents
Pericardial effusion	Acute tachypnoea Hepatomegaly Dullness extending beyond apex beat Decreased heart sounds	Difficult to diagnose clinically unless severe
Peripheral lymph node TB	Systemic signs/symptoms may be absent Mainly large, often visible and asymmetrical, firm to hard, painless nodes in neck region Nodes may be matted, may become fluctuant, and may ulcerate causing a sinus tract (scrofuloderma)	95% of lymph node TB is in the neck region, <1% generalised lymphadenopathy. Unilateral axillary lymphadenopathy ipsilateral to the BCG scar in infants usually indicate a BCG complication
TB meningitis (subtype of CNS TB)	Vomiting without diarrhoea Increased sleepiness, lethargy Headache Behavioural changes, including reduced playfulness Weakness, paralysis, other focal signs, or convulsions are often late signs if initial complaints were not correctly interpreted Signs of meningeal irritation (e.g., neck stiffness)	Meningitis with 1–2 week-long prodrome of non-specific signs of illness, such as fever and lethargy. A small percentage of patients never have fever. Repeated visits to healthcare facility with same complaints a warning sign. History of contact with TB case important additional information to make early diagnosis
Tuberculoma (subtype of CNS TB)	Headache Vomiting without diarrhoea Behavioural changes Weakness, paralysis, or convulsions Ataxia	May occur in the presence or absence of TB meningitis. May also appear as a manifestation of IRIS during treatment for TB meningitis
Miliary TB	Tachypnoea Hepatosplenomegaly Crackles in lungs (uncommon)	Signs may be very subtle and usually diagnosed by CXR, although the typical miliary findings may occur late in the course. Commonly associated with TB meningitis
Osteoarticular TB	Chronic back pain (spinal TB) or joint pain Gibbus/lump on back Joint pain, swelling, erythema Cold abscesses in inguinal area or lower back and buttocks may be from psoas abscess breaking through from spinal TB	Often history of injury which does not improve. Spine most common location of osteoarticular TB (50%). Affected joints are mainly single weight bearing joints
Abdominal TB	Acute or chronic abdominal pain Abdominal distension usually non-tender Abdominal masses	Peritoneal TB is the most common subtype. Imaging findings vary based on which organ is involved
Ear/mastoid TB	Chronic suppurative otitis media Swelling behind the ear often with lymph node enlargement	

BCG = bacille Calmette-Guérin; CNS = central nervous system; IRIS = immune reconstitution inflammatory syndrome; CXR = chest X-ray.

**Table 2:** Updated TB disease treatment recommendations for children and adolescents^9^,^10^

Recommendation	Age group	TB type and criteria or comment
4-month regimen 2HRZ(E)/2HR^74^*	≥3 months and ≥3 kg to <16 years	Non-severe PTB on chest X-ray Lymph node enlargement without airway compression/obstruction Non-cavitary disease confined to less than one lobe of the lungs and without a miliary pattern Uncomplicated TB pleural effusion Peripheral lymph node TB with no evidence of TB elsewhere
6-month regimen 2HRZ(E)/4HR*	All ages	PTB or EPTB that does not fit the criteria for non-severe TB Other forms of EPTB except TB meningitis and osteoarticular TB TB in all young infants <3 months or weighing <3 kg irrespective of severity
4-month regimen 2HPMfxZ/2HPMfx	≥12 years and ≥40 kg	PTB irrespective of severity
12-month regimen 2RHZE/10RH	All ages	Recommended by the WHO to treat TB meningitis, but the following caveats should be considered: The recommendation is not based on data showing greater effectiveness compared to other regimens^77^ Emerging data suggest that higher doses of R, in the range of 20–30 mg/kg, are needed to improve neurological outcomes^78^ Based on recent pharmacokinetic data, doses on the higher end of the dosing range should be considered for R, H and Z^79^ Some experts replace E with an agent with superior CNS penetration; Lfx 20 mg/kg once daily is a commonly used alternative Osteoarticular TB
6-month intensive regimen for TB meningitis 6RHZEth	All ages	TB meningitis Includes higher than standard dosages for RIF and INH Treatment is extended to 9 months for children and adolescents living with HIV^80^
Adjuvant corticosteroids (prednisone or dexamethasone)	All ages	For the following indications, prednisone 2 mg/kg/day, up to 60 mg/day (or dexamethasone 0.3 mg/kg/day, up to 9 mg/day), can be given for 4 weeks, followed by a gradual decrease in dose (taper) over 2–4 weeks before stopping: Initial 6–8 weeks of treatment for TB meningitis (strongly recommended) IRIS occurring in CNS TB (strongly recommended) IRIS associated with other forms of TB (can be considered) Endobronchial TB or intrathoracic lymphadenitis with symptomatic airway compression (should be considered) TB pericarditis (recommended by some experts)
Pyridoxine (B6)	All ages	Co-administered at a dose of 1–2 mg/kg/day, up to 25 mg/day, with INH to limit risk of neuropathy in children and adolescents with the following indications: Children living with HIV Malnourished children All infants All adolescents, especially those who are pregnant Children and adolescents receiving high-dose INH (15–20 mg/kg/day)

* For the 2HRZ(E)/2HR and 2HRZ(E)/4HR regimens, if there has been treatment interruption for ≥14 days during the intensive phase, the regimen should be restarted. For 2HRZ(E)/2HR, if the child or adolescent missed doses on ≥30 days (cumulative) during the continuation phase, treatment should be restarted from the beginning of the intensive phase. If the child or adolescent missed doses on <30 days, complete treatment by adding all missed doses. For 2HRZ(E)/4HR, if the child or adolescent missed doses on ≥60 days (cumulative) during the continuation phase, treatment should be restarted from the beginning of the intensive phase. If the child or adolescent missed doses on <60 days, complete treatment by adding all missed doses.^9^

H, INH = isoniazid; R, RIF = rifampicin; Z = pyrazinamide; E = ethambutol; PTB = pulmonary TB; EPTB = extrapulmonary TB; Mfx = moxifloxacin; P = rifapentine; CNS = central nervous system; Lfx = levofloxacin; Eth = ethionamide; IRIS, immune reconstitution inflammatory syndrome.

The diagnostic process includes obtaining a sample for microbiological confirmation when resources allow. However, if microbiological assays are not available, or results are not returned in a timely manner, a clinical diagnostic evaluation should be carried out and treatment initiated if indicated.^10^ Microbiological confirmation, although ideal, is achieved in a low percentage of children and young adolescents. Older children and adolescents should be able to spontaneously expectorate sputum. For those who lack this ability, alternative specimens include gastric aspirate, induced sputum, stool and nasopharyngeal aspirate. Greater specimen volume and a combination of different sample types increase the diagnostic yield.^24^

An increasing number of WHO-approved rapid diagnostic tests can be performed on a wide range of samples, including stool. These assays can be used at primary and secondary care settings; are more sensitive and specific than smear microscopy, but less sensitive than culture; and provide a more rapid test of drug resistance than culture and phenotypic drug susceptibility testing (DST).^9^,^10^ Xpert^®^ MTB/RIF, Xpert^®^ MTB/RIF Ultra (Cepheid, Sunnyvale, CA, USA), Truenat^®^ MTB, Truenat^®^ MTB Plus and Truenat^®^ MTB-RIF Dx (Molbio Diagnostics, Verna, India) are the most commonly evaluated and used in children.^31^ Xpert^®^ MTB/XDR (Cepheid) is particularly useful in settings with high prevalence of drug-resistant TB. Lipoarabinomannan (LAM) tests detect the LAM antigen in urine and are useful for rapid diagnosis in immunocompromised children and adolescents; however, sensitivity is suboptimal in immunocompetent individuals, and the tests do not evaluate drug resistance.^24^ A recent article provides an in-depth review of sample collection methods and microbiological assays.^24^

Microbiological testing, i.e., mycobacterial culture and nucleic-acid amplification tests (NAATs), can confirm diagnosis in samples from EPTB sites, such as fine-needle aspiration biopsies from peripheral lymph nodes.^32^ Although diagnostic yield from other sites, such as cerebrospinal fluid, is suboptimal, sampling should always be attempted, when clinically indicated. In general, diagnostic yield is higher from culture of serosal membranes (e.g., peritoneum, pleura) compared to the surrounding serosal fluid (e.g., ascitic fluid, pleural fluid).^33^,^34^ Furthermore, when tissue biopsy is available, visualisation of histopathologic findings compatible with TB on tissue examination, such as necrotising granulomas, can support the diagnosis, but does not exclude other causes such as non-tuberculous mycobacterial disease.

The WHO has published new treatment decision algorithms and scoring for use in settings with and without CXR; these algorithms are based on diagnostic and treatment outcome data in children aged under 10 years presenting for evaluation of PTB in high TB burden settings.^9^ Although treatment decision algorithms may be helpful, they have yet to be validated in most settings. Follow-up evaluation of patients with presumed TB, irrespective of whether they are started on TB treatment, remains important to establish symptom resolution or reconsider diagnosis.

Drug-resistant TB should be considered in children and adolescents with clinical TB disease with known exposure to a person who has confirmed or presumed drug-resistant TB, or who has died or failed treatment for TB, or when the child or adolescent responds unsatisfactorily to treatment for drug-susceptible TB despite good adherence. The DST results of the likely source case should always be considered in making therapeutic decisions until the child or adolescent’s microbiological results are available. Separate clinical standards for drug-resistant TB will be developed.

## STANDARD 3

**TB treatment initiation is particularly urgent in children and adolescents with presumptive TB meningitis and disseminated (miliary) TB.**

TBM and disseminated (miliary) TB, which is associated with a high risk of central nervous system (CNS) TB, pose a grave risk of rapid disease progression with mortality or irreversible neurological sequelae.^35^ Studies from a range of settings consistently show associations between delayed diagnosis and treatment and worse outcomes.^36^–^40^ Clinicians must maintain a low threshold for evaluating and empirically treating TBM. Young children, especially infants and immunocompromised individuals, are at greatest risk of disseminated TB and CNS TB, and may present with non-specific symptoms and signs that do not localise to the CNS, especially in the early stages of TBM.^41^,^42^ Careful consideration should be given to possible CNS involvement in any young child or immunocompromised individual with confirmed or clinically diagnosed TB, especially if there are signs of disseminated TB, and even in the absence of overt neurological symptoms or signs (Table 1). Infants with disseminated TB should receive a lumbar puncture. Some experts in well-resourced settings recommend lumbar puncture on all infants with microbiologically confirmed or clinically diagnosed TB disease to evaluate for TBM.^43^

Microbiological confirmation should be sought but should not delay treatment initiation. Whenever possible, cerebrospinal fluid (CSF) should be obtained and sent for Xpert Ultra testing or another NAAT, as well as mycobacterial culture, cell count, protein and glucose. Although CSF culture and NAAT have low sensitivity for TBM,^36^,^44^ CSF findings (typically, leukocytosis with a lymphocytic predominance, high protein and low glucose) and imaging findings (especially basal meningeal enhancement, which may be accompanied by hydrocephalus and/or infarcts) often help distinguish TB from other causes of meningitis or meningoencephalitis. CSF abnormalities may be less pronounced early in the course of TBM, and lumbar puncture should be repeated if diagnostic uncertainty persists. TB treatment and corticosteroids (as shown in Table 2) should be started in any child with TBM (clinical or based on CSF pleocytosis) who has no immediate evidence of another cause (positive Gram stain, NAAT, or culture), and who has evidence of any of the following: 1) hydrocephalus (clinical or radiographic); 2) vascular compromise or stroke (clinical or radiographic); 3) cranial nerve abnormalities; 4) basal meningeal enhancement on imaging; or 5) recent contact with a person with TB.

In patients with CNS TB, including TBM, living with HIV and not currently on antiretroviral therapy (ART), the WHO recommends postponing ART initiation to 4–8 weeks after the start of TB treatment to reduce the risk of CNS IRIS (immune reconstitution inflammatory syndrome).^9^,^45^ IRIS in CNS TB in patients living with HIV may be life-threatening and should be treated with corticosteroids (Table 2). Decisions about ART initiation, suspension and reintroduction should be made in consultation with a paediatric HIV expert whenever possible.

## STANDARD 4

**Children and adolescents with TB disease should be treated with an appropriate weight-based regimen as recommended by the WHO and/or national guidelines.**

Prompt treatment using an effective regimen, with optimal dosages and good adherence, is imperative for a successful outcome. Treatment initiation is urgent for severe forms of PTB (i.e., with extensive lung involvement and/or causing respiratory compromise), as these forms also pose a high risk of mortality and morbidity. ART should be initiated within the first 2 weeks of TB treatment for children and adolescents living with HIV who are not already on ART, with the exception of those with CNS TB (see Standard 3).^9^,^45^ Recommendations for managing drug-drug interactions, including dose adjustments for ART agents, are given elsewhere.^46^ The WHO recently updated treatment recommendations, including shorter regimens for children and adolescents with non-severe disease (Table 2).^10^ Rifampicin (RIF), isoniazid and pyrazinamide in the intensive phase, followed by RIF and isoniazid in the continuation phase, remain standard treatment for confirmed or presumed drug-susceptible TB in children. Ethambutol is recommended as the fourth drug in the intensive phase to treat PTB with high bacillary load, such as cavitary TB; for severe forms of EPTB; and for patients living with HIV. At the recommended doses, the risk of optic neuropathy due to ethambutol is negligible in children with normal renal function; thus, this drug can be given in all ages regardless of ability to cooperate with vision testing.^47^ When a choice of recommended regimens is available, the regimen should be selected in consultation with the caregiver and the child or adolescent as developmentally appropriate.^48^ Comorbidities, drug allergies, concomitant medications and, in adolescents, contraception and substance use, must be reviewed to avoid adverse drug effects and drug–drug interactions. RIF and other rifamycins interact with hormonal contraceptives and many other drugs, including antiretrovirals.^7^ Adolescents should be encouraged to abstain from alcohol during treatment, especially during the intensive phase.

Dosages of drugs are determined by weight (Table 3).^9^ First-line drugs are available in dispersible fixed-dose combinations that facilitate treatment adherence in children, but not all countries have access to these child-friendly formulations.^49^,^50^ Treatment response should always be monitored by clinical assessment, including weight. The need for radiological and microbiological assessment during treatment follow-up is required in those with severe or complicated disease, clinical deterioration or lack of treatment response despite good adherence. However, weight gain and symptom resolution indicate clinical improvement and are usually sufficient to confirm treatment response;^51^ weight increase may require dosage adjustment. Routine laboratory monitoring for adverse treatment effects is generally unnecessary, unless there is existing liver disease or concomitant use of other potentially hepatotoxic medications.^9^,^52^ The WHO Operational Handbook Module 5 provides further guidance on evaluating and managing hepatotoxicity in children and adolescents on TB treatment.^9^ Poor treatment response may be due to suboptimal adherence, incorrect dose calculation, frequent vomiting, poor gastrointestinal absorption of medications (in which therapeutic drug monitoring may assist where available^53^), drug resistance or an alternative diagnosis. Children and adolescents with and without HIV infection may experience IRIS, which presents as clinical worsening after an initial period of improvement, although IRIS is a diagnosis of exclusion. Severe IRIS can be managed with a course of systemic corticosteroids (Table 2).^54^

**Table 3:** Recommended doses for treatment of drug-susceptible TB disease*^9^,^80^

Medicine	Dosing range (maximum dose)	Dosing range for 6HRZEth TB meningitis regimen (maximum dose)	Dose for 2HPMfxZ/2HPMfx^†^
INH	7–15 mg/kg (300 mg)	15–20 mg/kg (400 mg)	300 mg
RIF	10–20 mg/kg (600 mg)	22.5–30 mg/kg (600 mg)	
PZA	30–40 mg/kg (2,000 mg)	35–45 mg/kg (2,000 mg)	1,500–1,600 mg; 2000 mg for ≥65 kg
EMB	15–25 mg/kg (1,600 mg)^81^		
ETH		17.5–22.5 mg/kg (750 mg)	
RPT^†^			1,200 mg
MFX^†^			400 mg

* All medications are dosed once daily.

† For adolescents ≥12 years old and ≥40 kg.

H, INH = isoniazid; R, RIF = rifampicin; Z; PZA = pyrazinamide; Eth = ethionamide; P, RPT = rifapentine; Mfx = moxifloxacin; EMB = ethambutol.

## STANDARD 5

**Evaluation and treatment of TBI is important to prevent disease, especially for those at high risk of disease progression following infection.**

Established risk factors for progression to disease should be considered to determine the urgency of the evaluation and initiation of treatment of TBI. Detailed standards for the diagnosis, prevention and treatment of TBI were published recently.^8^ Any child or adolescent with documented infection, without evidence of TB disease, should be treated to prevent progression to TB disease currently or in the future. The highest risk of TBI progressing rapidly to disease is in young children and those with immunocompromising conditions. The efficient screening and management of household or close contacts of TB patients should be routinely and promptly implemented, especially when the index patient has microbiologically confirmed PTB. Contact tracing and management achieves 1) the early detection and treatment of TB disease, and 2) the provision of treatment for documented or highly possible TBI, commonly referred to as TPT, in contacts who do not have TB disease at the time of assessment. Others who require evaluation for TBI include children and adolescents about to be treated with immunosuppressive drugs; those with immunocompromising conditions, including HIV infection and significant malnutrition; and neonates born to mothers with TB in pregnancy or the perinatal period.

As mentioned in Standard 2, tests of TBI include the traditional TST, IGRAs and newer TBSTs. The latter two types of tests are more specific for TBI than the TST.^29^ False-positive results are possible with all tests, but the positive predictive value is high if the patient has known recent TB exposure. Tests of TBI can remain negative for 8–12 weeks after inhalation of the organisms in immunocompetent individuals. As young children, especially infants, and immunocompromised children may develop TB disease during this window of time, they should be initiated on TPT even if the initial test of infection is negative.^55^ If a second test of infection performed 8–12 weeks after TB exposure has stopped (by separation from or effective treatment of the source person) is negative, treatment can be discontinued; if the second test is positive, a full course of TPT should be completed. If tests of TBI are not available, a full course of TPT should be given to all asymptomatic children and adolescents following known exposure to an individual with infectious PTB. A range of regimens is recommended to treat TBI in children and adolescents (Table 4).^9^,^49^ The choice of regimen will depend on national guidelines, age and weight, availability of suitable formulations, patient preference, and other comorbidities and concomitant medications, including ART. In general, shorter regimens are preferred to improve adherence. The incidence of severe adverse effects (especially hepatitis) from these regimens is low in children and adolescents; routine laboratory monitoring is discouraged unless the child has known or presumed liver disease or is taking other potentially hepatotoxic medications.

**Table 4:** Regimen options to treat TB infection in children and adolescents^9^

Regimen		Expected number of doses	80% of expected doses	Acceptable timeframe for TPT completion	Specific considerations or population	Management of treatment interruptions
6H (or 9H)	6 (or 9) months of daily INH 10–15 mg/kg (maximum 300 mg)	6H: 182 9H: 270	6H: 146 9H: 216	6H: 239 days* 9H: 360 days*	Standard traditional regimen but lower completion and safety profile than the shorter regimens below Suitable for children living with HIV and receiving ART, although shorter regimens preferred for adolescents^†^ 9H is used instead of 6H in the United States	For interruptions <2 weeks, finish the remaining doses For interruptions ≥2 weeks If ≥80% of expected doses have been taken, finish the remaining doses If <80% of expected doses have been taken and the entire regimen can be completed within the acceptable timeframe (see relevant column), finish the remaining doses If <80% of expected doses have been taken and the regimen cannot be completed within the acceptable timeframe, restart TPT
3HR	3 months of daily RIF 10–20 mg/kg (maximum 600 mg) and INH 10–15 mg/kg (maximum 300 mg)	84	68	120 days*	Suitable for HIV-negative young child (<5 years) contacts using dispersible fixed-dose combination of HR 75/50 mg	
4R	4 months of daily RIF 10–20 mg/kg (maximum 600 mg)	120	96	160 days*	Suitable for HIV-negative children and adolescents if suitable single RIF formulation available Liquid formulation not recommended (poor bioavailability), but capsules can be opened, and sprinkles mixed with food or water for administration to young children	
3HP	3 months of weekly (12-dose) INH and RPT^‡^	12	Irrelevant for managing treatment interruptions	16 weeks	Preferred regimen for adolescents living with HIV if on TDF, EFV, DTG or RAL-based ART Not yet recommended for children <2 years old Large pill burden and currently available preparations make use challenging for young children^§^	For 1 missed dose If remembered within the next 2 days, take the missed dose immediately and continue the schedule for weekly intake as originally planned If remembered >2 days later, take the missed dose immediately and change the schedule for weekly intake to the day the missed dose was taken until regimen completion (to avoid taking 2 weekly doses ≤4 days apart) If unable to complete weekly doses in ≤16 weeks, restart TPT
1HP	One month of daily RPT 600 mg and INH 300 mg	28	23	6 weeks or 8 weeks (see last column)	Not currently recommended for children or adolescents <13 years old Can be given to adolescents ≥13 years old living with HIV if on TDF, EFV, DTG, or RAL-based ART	For interruptions <7 days If ≥80% of expected doses have been taken, finish the remaining doses If <80% of expected doses have been taken, finish the remaining doses in ≤6 weeks For interruptions ≥7 cumulative but not consecutive days, finish the remaining doses and complete the regimen in ≤8 weeks For interruptions ≥7 consecutive days, restart TPT

* Acceptable timeframe for treatment completion is defined as the originally planned treatment duration +33% additional time in days.

† Rifamycin-containing regimens should be used with caution in children and adolescents living with HIV and on antiretroviral therapy because of potential drug–drug interactions. They can be used with EFV-based ART regimens.^9^,^46^

‡ INH and RPT dosing for ages 2–14 years: 10 to <16 kg, H 300 mg/P 300 mg; 16 to <24 kg, H 500 mg/P 450 mg; 24 to <31 kg, H 600 mg/P 600 mg; ≥31 kg, H 700 mg/P 750 mg. Dosing for ages ≥15 years: H 900 mg/P 900 mg.

§ May change as recommended dosage data and child-friendly preparation of RPT (150 mg) become available.

H, INH = isoniazid; ART = antiretroviral therapy; R, RIF = rifampicin; TPT = TB preventive therapy; P, RPT = rifapentine; TDF = tenofovir disoproxil fumarate; EFV = efavirenz; DTG = dolutegravir; RAL = raltegravir.

## STANDARD 6

**Children and adolescents treated for *M. tuberculosis* infection or TB disease should receive home-based/community-based treatment support whenever possible.**

Home-based treatment support should be provided for the full duration of treatment for TB disease or TPT. Poor adherence is a particular problem in TPT provision, and education and treatment support are essential to ensure completion. Inpatient care is indicated in some children and adolescents, such as those with severe TB disease until they are clinically stable, or those who need specific investigations to confirm the diagnosis. In such circumstances, most patients can be discharged for outpatient care soon after treatment initiation. Children and adolescents should not be hospitalised to ensure treatment adherence, unless no reliable caregiver or treatment supporter can be identified.^56^ In resource-limited settings, treatment may be initiated and supported to completion at the primary care level. Decentralised models of care for detection and treatment are supported by the recent WHO recommendations, and these models have demonstrated improved treatment outcomes, in addition to increased case detection and uptake of TPT.^10^,^57^ Home-based or community-based support for treatment of disease and infection that is coordinated with the facility-based clinic staff is much preferred over hospital/facility-based treatment support. Weight monitoring is important for following up treatment response and to make dose adjustments, especially in young children. However, frequent face-to-face follow-up appointments at the facility for review or to collect medication are usually not required and cause unnecessary costs to families and health services, as well as unnecessary disruptions to education or employment.^58^ Alternative ways to effectively follow-up at home (e.g., video-supported treatment using smartphone applications, telemedicine portals), which have developed further during the COVID-19 pandemic, should be considered.^59^

Children with TB rarely require isolation, and even those at risk of transmitting *M. tuberculosis* will be rendered non-infectious within days of treatment initiation. Most children and adolescents with infectious TB should be allowed back to school or work (without a mask) after 2 weeks of treatment if they are adherent to and tolerating an effective regimen and showing clinical improvement. Adolescents and, rarely, children with extensive disease – such as lung cavitation and/or extensive bilateral infiltrates – may require a longer period of isolation.^60^

## STANDARD 7

**Children, adolescents and their families should be provided adequate, age-appropriate support to optimise engagement in care, treatment adherence, clinical outcome, and the detection and management of adverse drug reactions and post-TB sequelae.**

To optimise engagement in treatment of children and adolescents as well as their families/caregivers, care must be provided in a friendly, non-judgmental, culturally sensitive manner with attention to health literacy levels and preferred language.^61^,^62^ Adolescents with TB require even more nuanced and personalised care, as discussed elsewhere.^3^,^26^ For treatment of both TBI and TB disease, a responsible caregiver (such as a family member or community health worker) should be identified to support each child or adolescent throughout treatment. The caregiver should engage with healthcare providers to ensure medication availability, treatment adherence and appropriate management of any drug-related adverse events.^9^ Establishing strong collaborations among the patient, the family, the community healthcare provider and the facility-based providers is critical from treatment initiation through to completion. Clinics that can counsel, support and care for all members of the TB-affected family regardless of age (adults, adolescents and children) or TB status (exposed, infected or ill) may improve the care experience and limit the number of appointments and integrate needs. Decentralisation of child TB services is recommended and will facilitate access to and integration of care.^10^,^57^ Diagnosis and treatment at healthcare facilities should be accessible (e.g., free access to TB medications vs. fee-for-medications, no medication stock outs). Using developmentally appropriate terminology, adequate education and counselling must be provided to children, adolescents and their caregivers at the time of diagnosis and with each follow-up visit.^63^,^64^ Adolescents should be involved in their own care, be encouraged to ask questions, and allowed to voice concerns to a healthcare provider in a confidential manner.^3^

Education and counselling to support initiation and completion of treatment is critical. Education is particularly important for initiation and adherence to TPT, as it is challenging for families and community healthcare workers to understand that a well child or adolescent requires treatment. With respect to TB disease, the importance of adherence for the entirety of treatment, despite symptom improvement or resolution, must be emphasised repeatedly. Possible adverse treatment effects and their management should be discussed in advance, as adverse effects may lead to poor adherence.^65^,^66^ Incentives, enablers, reminders, tracers and electronic medication monitoring boxes have shown some value in improving adherence and could be implemented where feasible.^64^ Families of children and adolescents with disease need to understand when to seek advice as regards clinical deterioration and have a clear follow-up plan that best suits their needs as regards timing and setting. As TB often occurs among the most socio-economically disadvantaged people, nutritional evaluation and food security should be assessed and nutritional support provided, whenever possible. Furthermore, families will often need financial support to cover medicine (if not free in public sector health system) and/or travel expenses. Some countries have a social grant system to support families, including child-care grants for children with permanent disability. Families may require guidance to access these resources. When resources are available, adolescents on TB treatment should be screened for depression,^3^ and both children and adolescents should be referred for mental health support as needed and where available. Issues regarding stigma experienced at a personal, family or community level should also be addressed, for example, by providing correct information about TB to families and communities.^28^

At the end of TB treatment, most children and adolescents require clinical assessment only. If clinically indicated, such as due to severe disease or ongoing symptoms, radiological and/or microbiological reassessment may be performed. Children and adolescents with severe PTB, who remain symptomatic at the end of treatment, have bronchiectasis or other extensive lung destruction on imaging or are living with HIV should be evaluated for post-TB lung disease (PTLD), defined as “evidence of chronic respiratory abnormality, with or without symptoms, attributable at least in part to previous (pulmonary) TB.”^5^,^46^ PTLD is the focus of another IJTLD Clinical Standards for Lung Health.^5^ Children and adolescents with spinal TB should be evaluated at least annually until they reach skeletal maturity to check for potential progressive deformity, which could lead to neurological, cardiopulmonary, orthopaedic and psychological complications.^67^ Children and adolescents with TBM should be assessed throughout treatment and thereafter, and referred as early as possible for multidisciplinary care to manage sequelae, including feeding issues, mobility issues, hearing and vision loss, neurocognitive impacts and endocrinopathies.^68^ They should also receive ongoing educational evaluation and appropriate educational support.

## STANDARD 8

**Case notification by health facilities, reporting and recording by in-country health authorities to the WHO, and contact tracing should be conducted for each child and adolescent with TB.**

Of the estimated 1.1 million children and young adolescents (ages 0–14 years) who developed TB disease in 2021, only 448,000 were notified to the WHO.^2^ The large number of missing patients may be due to under-diagnosis, but many may also be due to under-reporting. Better reporting of child TB is urgently required to improve our understanding of the magnitude of cases and to improve advocacy and public health strategy. Every child and adolescent who is diagnosed with TB at any health facility, including inpatient facilities and those in the private sector, must be notified to the national TB programme (NTP) or equivalent health authority, at diagnosis. The following data should be recorded for each patient: age, sex, site of disease, test results (acid-fast bacilli smear, NAAT, culture, DST), HIV status, other comorbidities, treatment dates (start, end, interruptions) and treatment outcome. The WHO has requested that NTPs disaggregate paediatric data by 5-year age bands (0–4, 5–9, 10–14 and 15–19 years).^69^ Further disaggregation of EPTB data by disease site, particularly CNS, also is important.

Contact tracing contributes to case detection and provision of TPT. Children and adolescents are not only the household contacts but, when they have infectious forms of TB, also can be the source of infection (especially adolescents). All contacts, both household and non-household (e.g., school, daycare, work, social settings), of these children and adolescents should be evaluated. Reverse contact tracing should be performed to identify the likely source of infection and to provide appropriate treatment.^70^ Documentation of contact screening and management should be made and reported to the NTP. All TPT provision, including coverage of eligible groups, as well as uptake and completion of TPT, must be recorded and reported to the NTP.^8^

## FUTURE RESEARCH PRIORITIES

Over the past 20 years, childhood TB has gained increasing recognition and prioritisation by WHO, NTPs and other public health institutions. Accordingly, substantial progress has been made in childhood TB research, from improved estimates of the global incidence and mortality to a shorter effective regimen to treat non-severe TB disease in children.^4^,^71^–^74^ In contrast, adolescent TB remains relatively neglected.^75^ Many research gaps remain for both children and adolescents,^76^ which we have categorised as follows:

### Epidemiology

- Improve estimates of adolescent TB incidence and outcomes, including loss from treatment and mortality;
- Understand the burden – including incidence of disease and sequelae – of distinct forms of EPTB, particularly CNS TB and other severe forms associated with poor outcomes;
- Estimate the prevalence of PTLD in children and adolescents;
- Characterise the co-occurrence of mental health disorders, including depression and anxiety, among older children and adolescents with TB;
- Evaluate completeness of TB case recording and reporting of both treatment for TB disease and TBI.

### Prevention

- Assess practical methods to optimise the full TPT cascade of care (identifying contacts, excluding TB disease, starting TPT and completing TPT);
- Evaluate shorter TPT regimens, including drug dosing and safety, especially in the context of drug-resistant TB exposure and in children <2 years of age;
- Develop a more effective vaccine against TB disease for use in infants and at all ages.

### Diagnosis

- Develop tests that can predict risk of progression to TB disease;
- Develop more sensitive screening tests that can be used for TB triage;
- Develop tests that can distinguish between TBI and TB disease;
- Develop more accurate tests to confirm TB disease from respiratory and non-respiratory samples;
- Validate the WHO treatment decision algorithm in diverse settings;
- Develop and validate computer-aided reading of digital CXR for young children and improve the accuracy of this technology for older children and adolescents.

### Treatment

- Conduct more timely dosing and safety studies of new drugs in children and adolescents before their licensing;
- Conduct treatment shortening studies in children, and include adolescents in adult TB treatment shortening studies;
- Optimize treatment for TBM, including adjuvant anti-inflammatory therapies;
- Optimize treatment of corticosteroid-refractory, paradoxical inflammatory reactions in CNS TB.

### Operational research

- Better understand barriers, preferences, and needs with respect to TB diagnosis, treatment, and treatment retention among children, adolescents, and their families;
- Identify best practices to promote earlier diagnosis of TB;
- Determine the most appropriate and cost-effective TB service delivery models for children and adolescents;
- Develop and evaluate strategies to improve treatment adherence and engagement in adolescents;
- Evaluate programme integration strategies for paediatric TB, including with HIV, maternal, neonatal and child health, adolescent health, nutrition, and other relevant programs;
- Develop programmes to reduce stigma among children and adolescents and their families affected by TB.

## CONCLUSION

Despite being preventable and curable, TB remains a major global cause of mortality and morbidity in children and adolescents. TB can lead to long-term physical, psychological, social and economic sequelae for patients and their families. These standards reflect evidence-based and universally recommended approaches to detect, treat and prevent TB in children and adolescents. However, a wide policy–practice gap remains, especially in the provision of TB services for children and adolescents in TB-endemic countries, where the most vulnerable populations reside. These clinical standards, which incorporate the latest available evidence, as well as expert consensus when data are not available, aim to close this policy–practice gap and to optimise child-, adolescent- and family-centred TB care and prevention.
